# Piezosurgery versus conventional rotary surgery for impacted third molars: A randomised, split-mouth, clinical pilot trial

**DOI:** 10.4317/medoral.25929

**Published:** 2023-11-22

**Authors:** Ahmet Demirci, Ferit Bayram, Guhan Dergin

**Affiliations:** 1 DDS, Faculty of Dentistry, Department of Maxillofacial Surgery, Marmara University, Istanbul, Turkey; 2 DDS, PhD, Faculty of Dentistry, Department of Maxillofacial Surgery, Marmara University, Istanbul, Turkey; 3 DDS, PhD, Faculty of Dentistry, Department of Maxillofacial Surgery, Marmara University, Istanbul, Turkey

## Abstract

**Background:**

Few studies have compared the effects of piezosurgery and conventional rotary surgery for impacted wisdom teeth on the quality of life. Among these studies, the inclusion parameters and evaluation methods have varied.

**Material and Methods:**

This study aimed to compare the effects of piezosurgery and conventional rotary instruments on the quality of life using a standardised method. Patients with bilateral and symmetric mandibular impacted wisdom teeth were included based on the Winter and Pell-Gregory scale and Yuasa difficulty index criteria. The primary objective was to assess the effects of the methods on the quality of life using the Oral Health Impact Profile-14 questionnaire. Secondary objectives included comparisons of swelling, trismus, pain, and total operative times. The study was conducted between October 2021 and March 2022. The clinical trial protocol was recorded in the United States National Library of Medicine clinical trial registry (NCT05545553).

**Results:**

We enrolled 20 patients (40 wisdom teeth) and found that the removal of impacted teeth using the piezosurgery method positively affected the quality of life and considerably improved swelling, trismus, and pain scores. However, piezosurgery may affect postoperative morbidities such as increased total operative times.

**Conclusions:**

Piezosurgery appears to have advantages over conventional rotary surgery for impacted wisdom tooth extraction in terms of quality of life and postoperative symptoms. However, further research should investigate potential drawbacks and confirm these findings.

** Key words:**Third molar, piezosurgery, osteotomy, pain, quality of life, swelling, trismus.

## Introduction

Approximately 2,000 randomised, controlled trials of tooth extraction exist; however, minimising related surgical complications and symptoms remains a topic of interest. Currently, clinicians recommend new flap designs (1), antimicrobial mouthwash (2), preventive antibiotics (3), corticosteroids (4), drainage (5), rotary instruments at different speeds (6), electromagnetic devices or osteotomes (7), coronectomy (8) and piezosurgery (9) to reduce postoperative morbidity. Studies have indicated that piezosurgery reduces postoperative sequelae, thus improving the quality of life; therefore, it is recommended if the teeth have particularly dangerous or unusual positions (10).

Studies including meta-analyses have compared piezosurgery and conventional rotary surgical methods for impacted wisdom teeth (9,11,12). Generally, they concluded that piezosurgery reduces postoperative pain and trismus. However, differing evaluation methods among these studies have made it impossible to draw conclusions about the amount of swelling and neurosensory damage (13,14). Moreover, few studies have examined the effects of these techniques on the quality of life, and closer inspection of these studies revealed that they were not designed to be split-mouth (15), used a scale to determine the angulation and depth of the teeth to be included in the study (12) and used scales specific to chronic disease groups, such as the quality of life scale (16). Therefore, similar studies with more specific tests that examine the impact of oral health on the quality of life are needed.

During wisdom tooth extraction studies, parameters such as pain, swelling, and trismus can vary depending on demographic factors, such as the patient’s age, sex, and anxiety. However, split-mouth studies are recommended to reduce the effects of these factors on the results (12). Furthermore, the position of the teeth may influence the results; therefore, the selection of symmetrically similar teeth should provide more accurate results (17). Additionally, variables such as the piezosurgery tip and operator experience may affect the results and should be controlled during the study (18). Therefore, this randomised, controlled, split-mouth study investigated the effects of conventional rotary and piezosurgery surgical methods on the quality of life after angled mandibular impacted tooth extraction as well as pain, swelling, trismus, and the total operative time. We hypothesised that the quality of life would not differ between the two methods.

- Abbreviations

GO-AN: Distance between the mandible corner and the nose wing; GO-CA: Distance between the mandible corner and the lateral canthus of the eye; GO-CM: Distance between the mandible corner and the oral commissures; GO-POG: Distance between the mandible corner and pogonion; GO-TR: Distance between the mandible corner and tragus; OHIP-14: Oral Health Impact Profile-14.

## Material and Methods

- Study design

From October 2021 to March 2022, we enrolled patients who presented to our institution for the extraction of their impacted third-molar teeth. The clinical trial protocol, which adhered to the CONSORT guidelines (Table 1), was retrospectively recorded in the United States National Library of Medicine clinical trial registry (NCT05545553).

**Table 1:**
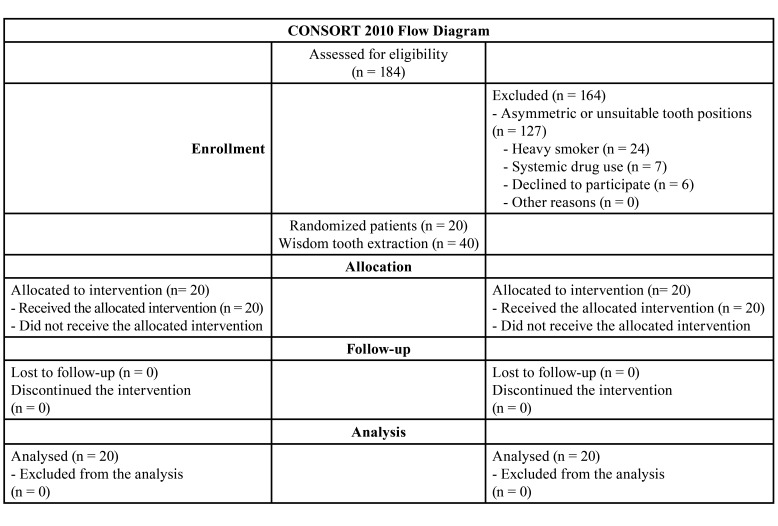
Study flowchart.

Prior to patient selection, the Marmara University Faculty of Medicine Clinical Research Ethics Committee evaluated and approved the study protocol (reference number: 09.2021.698) in accordance with the principles outlined in the Declaration of Helsinki for biomedical research involving human patients. All eligible patients were provided with comprehensive information about the pre-treatment process, surgical procedure, postoperative period, possible complications, and recovery time, and they gave their oral and written informed consent to participate in the study.

A similar study that examined the postoperative quality of life was used to determine the sample size. Piersanti *et al* (12) reported that the Posse values of the control and test groups were 36.0 ± 7.6 and 24.7 ± 10.3, respectively. Therefore, we predicted that at least 18 patients should be included in the study based on an impact strength of 1.26 and 95% power. Consequently, we aimed to enrol 20 patients (40 impacted teeth) because of the possibility of patient dropout during the study and follow-up period.

**Figure 1:**
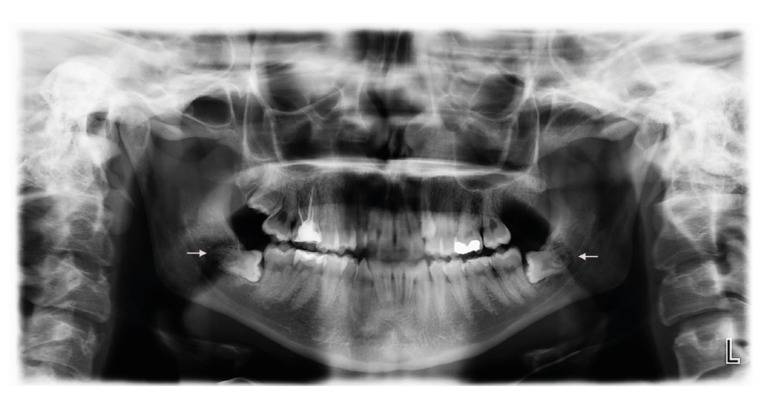
Orthopantomogram of a representative patient.

We included individuals with an American Society of Anesthesiologists systemic status of I or II who were 18 to 35 years of age and had asymptomatic mandibular third-molar teeth requiring prophylactic or orthodontic extraction. All teeth had symmetrical class 2, position B according to the Winter and Pell-Gregory classification and the same degree according to the Yuasa difficulty index (19) (Fig. 1). Patients were excluded if they had systemic disease affecting bone or soft tissue metabolism, smoked more than ten cigarettes per day or were alcohol-dependent, had acute pericoronitis or acute periodontal disease at the time of surgery, and used antibiotics for acute infection. The flowchart (Table 1) presents the participation protocol.

To randomise the study, we used a computer to randomly generate a number for each patient. If the number was odd, the patient received conventional bur surgery as the first procedure; however, if the number was even, the patient received piezosurgery as the first procedure and conventional surgery as the second procedure. A coin toss decided the first tooth extraction area (left or right). The patient and investigator who measured and evaluated the postoperative surgical outcomes were unaware of which technique was used. All procedures were performed by a single surgeon using the same surgical and sterilisation methods.

- Surgical procedure

**Figure 2:**
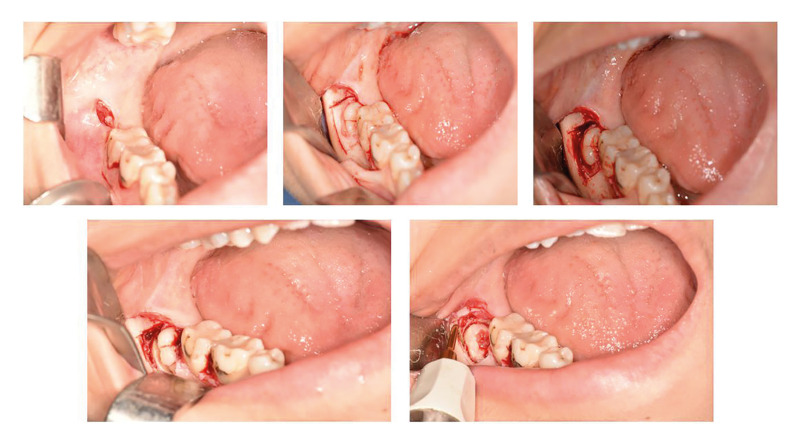
Osteotomy of the bone around the wisdom tooth using the piezosurgery device.

Before surgery, patients were administered 0.12% chlorhexidine gluconate for 1 min. Adequate anaesthesia was achieved with inferior alveolar and lingual nerve blocks (2 mL) and vestibular infiltration (1 mL) with articaine hydrochloride (4%) and a 1:100,000 concentration of epinephrine (Ultracaine DS; Sanofi-Aventis, Bridgewater, NJ, USA). An envelope flap with a relaxing distal vertical incision was used to reach the operative site. In the conventional rotary group, the full-thickness flap was lifted, and the bone on the buccal and distal surfaces of the tooth were removed with an HM1T-numbered round tungsten carbide bur (Meisinger, Neuss, Germany). The tooth was divided using the HM31 fissure tungsten carbide bur (Meisinger) and extracted using an elevator. This process was performed using the surgical handpiece S-11 straight tip (W&H Austria, Bürmoos, Austria) at 35,000 rpm. In the piezosurgery group, the device settings were adjusted as follows before starting the procedure: irrigation, 6; function, cortical; and light, auto. The frequency was between 28 and 36 kHz, and the micro-vibration amplitude was between 30 and 60 μm/s. After removing the bone surrounding the tooth with the OT-12 tip (Mectron, Lorento, Italy), the tooth fissure was divided using a bur. The periodontal ligament space around the remaining root part was enlarged using an EX-1 tip (Mectron); a cleavage area was then created for the elevator, and the tooth was extracted using an elevator (Fig. 2). In both groups, the bleeding in the surgical field was controlled, and the incisions were closed primarily using simple intermittent 3-0 silk sutures (Doğsan, Trabzon, Turkey). The total operative time ranged from the first incision to the last suture.

Postoperatively, amoxicillin (1,000 mg, twice per day; Largopen; Bilim Pharmaceuticals, Istanbul, Turkey) was prescribed for 7 days to prevent infection. Additionally, an antiseptic mouthwash (Chloroben; Drogsan İlaç, Ankara, Turkey) was prescribed, starting the day after surgery (three times daily for 7 days) to help maintain oral hygiene and promote healing. Paracetamol (500 mg, twice per day; Parol 500 mg Film Tablet; Atabay Kimya, Istanbul, Turkey) was prescribed as needed for pain management. Patients were followed up on postoperative days 3, 7, and 14; sutures were removed on postoperative day 7. The two surgical procedures were performed 1 month apart; therefore, the results of one did not affect those of the other.

- Variables

The Oral Health Impact Profile-14 (OHIP-14) questionnaire was utilised to evaluate the impact of surgery on the quality of life. The OHIP-14 is a validated questionnaire developed by Slade (20) in 1997 that was adapted to the Turkish language by Mumcu *et al* (21). The questionnaire consists of 14 questions that cover the following seven categories: functional limitations, physical pain, psychological discomfort, physical deficiency, psychological deficiency, social deficiency, and unfavourable situations. Each question is scored using a scale of 0 to 4, with higher scores indicating a poorer quality of life. Patients completed the OHIP-14 questionnaire both preoperatively and on postoperative day 14, and the difference between the scores before and after surgery was calculated and used for the analyses. Trismus was measured using the technique described by Neupert (22) which included measuring the mandible corner-tragus (GO-TR), mandible corner-lateral canthus of the eye (GO-CA), mandible corner-nose wing (GO-AN), mandible corner-oral commissures (GO-CM), and mandible corner-pogonion (GO-POG) with a tape measure. Measurements were performed preoperatively and on postoperative days 3 and 7. The distance between the mesio-incisal corners of the upper and lower central incisors was measured with a ruler when the mouth was maximally opened; measurements were performed preoperatively and on postoperative days 3 and 7.

Pain was assessed daily for 1 week postoperatively using the visual analogue scale, which ranges from no pain (score 0) to severe pain (score 10). Additionally, patients recorded on a piece of paper the number of pain relievers they used during the first postoperative week. The total operative time ranging from the first incision to the last suture was measured during the analysis.

- Statistical analyses

Data are presented as means, standard deviations, medians, minimums, maximums, frequencies, and ratios. The distribution of the variables was measured using the Kolmogorov-Smirnov test. Paired-sample t-tests and Wilcoxon signed-rank tests were performed to analyse dependent quantitative data. All analyses were performed using SPSS version 28.0 (IBM Corp., Armonk, NY, USA).

## Results

**Figure 3:**
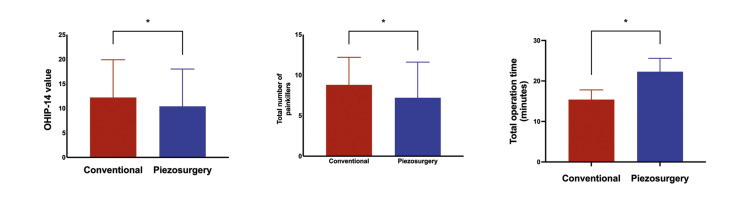
Oral Health Impact Profile-14 (OHIP-14) questionnaire score changes, total number of painkillers, and operative time comparisons. The OHIP-14 values represent the mean differences in the scores from baseline to postoperative day 14. *p < 0.05.

A total of 184 patients applied for study enrolment; however, we included only 20 (40 teeth; 11 men and 9 women; mean age, 22.4 ± 4.43 years). Of the included patients, none was later excluded or lost to follow-up. None of the patients experienced postoperative infection, bleeding, or nerve damage. However, the mean change in the OHIP-14 values was significantly higher in the conventional group (12.2 ± 7.7) than that in the piezosurgery group (10.4 ± 7.6; *p* < 0.05) (Fig. 3).

**Figure 4:**
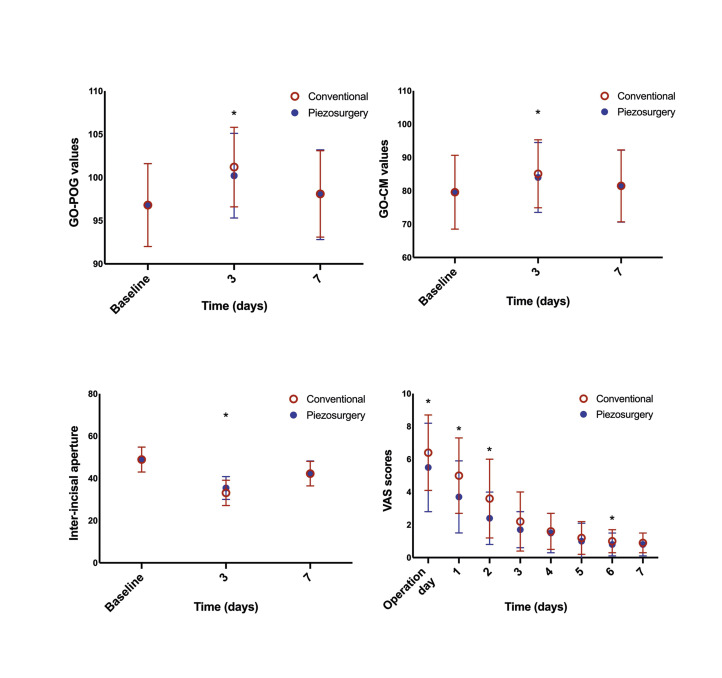
Comparisons of swelling, trismus, and visual analogue scale (VAS) scores of conventional surgery and piezosurgery. GO-CM: mandible corner oral-commissures; GO-POG: mandible corner-pogonion. *p < 0.05.

Measurements based on five reference points of the skin indicated significant differences only in the GO-POG and GO-CM values preoperatively and on postoperative day 3 (Fig. 4). The decrease between the preoperative value of maximum mouth opening and the postoperative 3rd and 7th day values were (13.5±5.4) mm and (6.6±4.8)mm in the piezosurgery group, and (15.9±6.1) mm and (6.8±4.2) mm in the conventional surgical group, respectively. Considering these values, although there was a significant difference in the decrease between the preoperative value and postoperative day 3 value of the maximum mouth opening for both groups, there was no significant difference between the preoperative value and postoperative day 7 value.

When the two methods were compared, the piezosurgery method resulted in a significantly smaller reduction in the mouth opening on postoperative day 3. However, there was no significant difference between the two methods on postoperative day 7 (Fig. 4). Both the piezosurgery and conventional rotary groups experienced the most pain on postoperative day 1. The piezosurgery method generally caused less pain during the postoperative period than did the conventional method. Furthermore, the pain level significantly differed between groups on the day of surgery (i.e., 12 hours after surgery) and postoperative days 1, 2, and 6, with VAS scores of (3.7±2.2, 2.4±1.6, 0.8±0.7) for the piezosurgery group and (5.0±2.3, 3.6±2.4, 1.0±0.7) for the conventional surgery group, respectively (Fig. 4). In addition, the total number of analgesics was significantly higher in the conventional surgery group (8.8 ± 3.4) than that in the piezosurgery group (7.2 ± 4.4; *p* < 0.05) (Fig. 3). Finally, the total operative time was significantly lower in the conventional surgery group (15.4 ± 2.4 min) than that in the piezosurgery group (22.3 ± 3.3 min; *p* < 0.05) (Fig. 3).

## Discussion

The results of this study showed that the piezosurgery method significantly improved the quality of life, reduced swelling, and decreased trismus compared to the conventional rotary method. However, it is worth noting that the total operative time was longer for piezosurgery. Although more surgeons are using the piezosurgery device for osteotomies in the maxillofacial region, the effectiveness and efficiency of the instrument remain debaTable. Because parameters generally evaluated by studies, such as pain, swelling, and trismus may vary depending on demographic factors (e.g. age, sex, and anxiety level), split-mouth studies likely provide more valuable data.

Wisdom tooth extraction surgeries can range from relatively easy to highly difficult (23) depending on the depth and angulation of the tooth and resistance of the surrounding bone. Consequently, the surgical difficulty affects postoperative morbidity. Only two of the 13 reported split-mouth studies standardised the depth and angulation of the teeth and degree of difficulty (17,24). Therefore, our study only included bilateral, class II, position B, impacted teeth because this type of impaction is common.

Impacted wisdom tooth surgery significantly affects the quality of life, and any surgical improvements contribute to patient satisfaction. We used the OHIP-14 scale to evaluate the quality of life of the two groups and found a significantly better score after using the piezosurgery method. During similar studies, Piersanti (12) and Goyal (15) used the postoperative symptom severity scale to assess the quality of life and reported similar results for piezosurgery. However, Menziletoglu *et al* (16) used the global quality of life scale and reported that, unlike our results, the quality of life did not significantly differ between groups.

During our study, the piezosurgery group had less pain, swelling, and trismus than did the conventional surgery group, indicating a better quality of life. Although the improved swelling and trismus scores observed in the piezosurgery group on postoperative days 1 and 3 disappeared by postoperative 7, the overall results were still better than those of the conventional group. Comparing postoperative morbidity results with those of other studies is challenging because many factors influence morbidity, such as the measurement methods and evaluation periods, amount of osteotomy performed in relation to the surgical difficulty, position of the teeth, type of incision, and how piezosurgery is performed throughout the procedure. When we compared our results with those of similarly designed studies, we found correlations with the results of the studies by Mozatti *et al* (24) and Arakji *et al* (17). However, our results differed from those of Menziletoğlu *et al* (16). This difference may be because of changes in the evaluation methods and timing. For example, we evaluated trismus on postoperative days 3 and 7. Although the difference between groups was statistically significant on day 3, this difference was not significant on day 7. Menziletoğlu, however, evaluated trismus only on postoperative day 7 and reported no significant difference between groups.

Perceived pain also affects the quality of life. There are no restrictions regarding postoperative analgesic use; therefore, we chose to use paracetamol, which has an analgesic effect on mild-to-moderate pain (25). In both groups, the most pain was reported on day 1, and it decreased over time. However, the pain level was significantly lower in the piezosurgery group on the day of surgery (i.e. 12 hours after surgery) and postoperative days 1, 2, and 6. These results are in agreement with those of studies with similar difficulty levels (17,24).

Similar to our study, Menziletoğlu *et al* (16) in a study on impacted third molars with the same Winter and Pell-Gregory classification reported that patients operated with conventional surgery had less postoperative pain because conventional surgery shortened the operation time, but this difference was not statistically significant. In our study, although the operation time of the patients who were operated on with piezosurgery was significantly longer than that of the patients who were operated on with a bur, the postoperative pain was less. The achievement of these results despite the prolonged operation time can be explained by several possible differences between the two methods. The most important part of third molar extraction is the bone removal process. Compared to conventional rotary instrument, the piezosurgery provided less injury of bone tissues, which insured a better blood supply resulting in lower incidence of postoperative inflammation (11). Keeping the tissue damage to a minimum in this process allows the patient to have a more comforTable recovery period. Although osteotomy is performed faster with burs surgery, osteonecrosis may occur because of thermal damage to the bone. Piezosurgery minimises the risk of thermal damage, thanks to the fact that it does not require much pressure during its use and that it has a continuous irrigation feature obtained with the microstreaming effect (15). Therefore, it ensures the significant recovery of the osseous reactions of the bone after the operation. The results of our study are consistent with those of recent systematic reviews and meta-analyses (9,11,14). It is also essential to consider that pain perception is subjective and can be influenced by factors like anxiety levels and individual pain tolerance.

Previous studies comparing piezosurgery and conventional rotary instruments for different procedures reported variable results, such as longer (26), shorter (27) or statistically insignificant differences (28) in operative times. We found that the operative time was significantly shorter in the conventional surgery group than that in the piezosurgery group, similar to other studies (15,29). When comparing our results to the meta-analysis by Liu *et al* (11) we found that our findings are generally consistent with their conclusions. They reported that piezosurgery resulted in less postoperative pain, swelling, and trismus compared to conventional rotary instruments. Based on our experience, burs are more comforTable for osteotomies around the tooth without a straight incision, especially during wisdom tooth surgery. Therefore, differences in our results and others may have occurred because no scale was used when classifying the amount of osteotomy and tooth position, which indirectly affect operative times.

Our study had some limitations. We used a two-dimensional metric measurement method because it is clinically practical and applicable for measuring swelling. However, some researchers argue that most swelling areas are outside the two-dimensional measurement areas; therefore, three-dimensional measurement methods should be used (30). Another limitation was that the piezo tip used for surgery differed from that used during other studies. Either the piezo tip type was not specified in previous studies or the tips in those studies were completely different from each other, and standardisation was not provided.

## Conclusions

Conventional surgery and piezosurgery methods have advantages and disadvantages, thus making it impossible to reach a definite conclusion with respect to one method being superior to the other. Piezosurgery reduces postoperative morbidity and improves the quality of life, but it prolongs the operative time.
